# Glycation resistance and life-history traits: lessons from non-conventional animal models

**DOI:** 10.1098/rsbl.2023.0601

**Published:** 2024-06-12

**Authors:** Cyrielle Duval, François Criscuolo, Fabrice Bertile

**Affiliations:** ^1^ University of Strasbourg, CNRS, Institut Pluridisciplinaire Hubert Curien, UMR 7178, Strasbourg 67000, France; ^2^ Infrastructure de Protéomique, ProFi, Strasbourg FR2048, France

**Keywords:** glycation, glucose resistance, longevity, reproduction, evolution

## Abstract

Glycation reactions play a key role in the senescence process and are involved in numerous age-related pathologies, such as diabetes complications or Alzheimer’s disease. As a result, past studies on glycation have mostly focused on human and laboratory animal models for medical purposes. Very little is known about glycation and its link to senescence in wild animal species. Yet, despite feeding on high-sugar diets, several bat and bird species are long-lived and seem to escape the toxic effects of high glycaemia. The study of these models could open new avenues both for understanding the mechanisms that coevolved with glycation resistance and for treating the damaging effects of glycations in humans. Our understanding of glycaemia’s correlation to proxies of animals’ pace of life is emerging in few wild species; however, virtually nothing is known about their resistance to glycation, nor on the relationship between glycation, species’ life-history traits and individual fitness. Our review summarizes the scarce current knowledge on the links between glycation and life-history traits in non-conventional animal models, highlighting the predominance of avian research. We also investigate some key molecular and physiological parameters involved in glycation regulation, which hold promise for future research on fitness and senescence of individuals.

## 1. Introduction

Since the observation of glycation reactions *in vivo* in living organisms [1], we have learned that glycation damage, notably through the formation of so-called advanced glycation end-products (AGEs), heavily contributes to senescence and ageing-related diseases [1,2]. As their name suggests, AGEs are produced late in the chain of reactions occurring during the glycation process, their particularity being that they are stable and, therefore, hardly degradable by the body. Consequently, they tend to accumulate in cells and tissues and can lead to altered protein conformation and functionality via the formation of protein aggregates. By binding to specific receptors, AGEs also promote oxidative stress and inflammatory reactions, thus playing a major role in the senescence process [3]. To date, most research has been conducted in human medicine, with the main aim of combating glycation’s deleterious health consequences.

Interestingly, some wild and/or non-conventional animal models do not seem to suffer from any of the known consequences of glycation on health. Despite their long lifespans, birds indeed usually express blood glucose levels similar to those observed in hyperglycaemic humans but without the expected deleterious effects [4]. A similar paradox can be found in nectarivorous and frugivorous bats, which are among the longest-lived mammals and do not seem to show any apparent signs of age-related diseases, despite relying on a high-sugar diet [5]. If glycaemia *per se* has been previously suggested to be correlated with proxies of species’ pace-of-life [6,7] or individual fitness [8] mainly based on energetic explanations, we hypothesize here that such a relationship also implies that evolution might have acted through the selection of glycation regulatory and/or resistance mechanisms. Resisting glucotoxicity via resistance to glycation, while glucose remains the main energy source in the environment, may well fall into a novel view of senescence evolution, not only based on trade-offs but also on the conjunction of structural, cellular, physiological or behavioural traits pushing further the limits imposed by physiological constraints, and so extending physiological homeostasis in life-threatening conditions [9].

Our primary objective here is to focus on non-conventional animal models and see what they can teach us in terms of (i) the links between glycation and life-history traits, at the intra- and interspecific levels, and (ii) the mechanisms that evolved to escape the deleterious effects of glycation (thereafter glycation stress). Because this knowledge remains in its infancy, we outline gaps and promote promising avenues of research, as they should enable us to better understand how glucose and glycation may support life-history traits’ evolution.

## 2. A few words on the glycation reaction and its known effects on health and senescence

The glycation reaction consists of the non-enzymatic binding of a reducing sugar to a free amino group of a protein. The unstable newly formed compound then undergoes a series of reactions, resulting in the formation of so-called Amadori compounds within a few days. The latter includes essential well-studied biomarkers such as glycated haemoglobin (HbA1c) or glycated albumin (GA). Those compounds can then undergo further irreversible reactions over a few weeks or months to finally form stable AGEs [10]. Because of their spontaneous character, rates of glycation reactions are expected to vary with blood glucose levels, but this idea is based on human medicine.

In biomedicine, senescence is characterized by the progressive deterioration of the structural integrity and function of cells and tissues over time, which leads to an increased risk of death. Lopez-Òtin *et al*. defined several major hallmarks of ageing [11] and, later, of health [12,13], among which glycation is likely to play a key role [3]. AGEs and Amadori compounds interfere in different physiological and cell mechanisms well known to alter senescence-related mechanisms, such as oxidative stress [14], chronic inflammation [15] or alteration of proteins’ structures and functionalities, which ultimately leads to ageing-related diseases [2]. More information on glycation’s contribution to the senescence process is given in table 1.

**Table 1.** Examples of glycation’s contribution to each hallmark of ageing (as defined by Lopez-Otìn *et al*. [3,11,15–24]).

hallmark of ageing (as defined by [11])	examples of glycations’ contribution
genome instability	glycation of nucleic acids and altered three-dimensional structure of DNA altered DNA repair mechanisms (because of impaired function of proteins and enzymatic pathways)
telomere attrition	accelerated shortening of telomeres (owing to DNA damage, increased ROS and pro-inflammatory metabolites production)
epigenetic alterations	incomplete degradation of CML in tumours
loss of proteostasis	glycation of amino acids and altered protein conformation impaired functionality of proteins deregulated protein clearance
disabled macroautophagy	altered protein three-dimensional structure and functionality
deregulated nutrient sensing	altered enzymatic pathways
mitochondrial dysfunction	altered respiratory function altered permeability of the membrane increased ROS production
cellular senescence	alteration of cellular membranes integrity (owing to glycated proteins and lipids) increased ROS production
stem cell exhaustion	abnormal activation or inhibition of Wnt/β-catenin signaling AGE–RAGE interaction abnormal differentiation of primary stem cells
altered intercellular communication	alteration of cellular membranes integrity (owing to glycated proteins and lipids) upregulation of RAGE pathways
chronic inflammation	upregulation of RAGE pathways increased ROS production increased production of pro-inflammatory metabolites
dysbiosis	change in gut microbiota’s composition (richness, diversity…) because of dietary AGEs

CML, N∈-(carboxymethyl)lysine; RAGE, receptor for advanced glycation end-products; ROS, reactive oxygen species.

In evolutionary biology, senescence (i.e. the decreasing fertility and increasing mortality rates with age [25]) stands as a key variable, as it has been shown to vary considerably between species [26] and individuals [27]. In this context, any senescence-related mechanism may have been the target of selection. How glycation stress covaries among and within species with contrasted pace-of-life or fitness, and under which genetic and environmental influences, remains an open question.

## 3. Exploring the links between glycation and life-history traits in non-conventional animal models

Evolutionary biologists consider that a trait is susceptible to natural selection if it meets the following conditions: (i) variability in its expression within a population, (ii) heritability and transmissibility between generations, and (iii) impact on fitness [28]. It has been demonstrated in humans that some glycation products (HbA1c, GA and carboxymethyl-lysine (CML)) meet the first two conditions [29]. Moreover, given that glycation levels are higher in patients suffering from ageing-related diseases (the latter increasing the risk of mortality), this suggests that glycation most likely has an impact on at least fitness-related traits. If such data concerning glycation are not yet available in animals other than humans, we, however, know that glycaemia is a trait that varies with life-history stage and environmental conditions [30]. Montoya *et al.* also showed that glucose regulation is a repeatable trait that is affected by handling in zebra finches (*Taeniopygia guttata*) [31]. This regulation capacity can be impaired by stress events, and it has been suggested that the coping ability of individuals to glucose-related physiological challenges could impact their fitness [32]. It, therefore, appears that glucose regulation may be a key physiological trait under selection [32–34]. As glycation rates are usually expected to be positively correlated with glycaemia [29,35], we might expect glycation levels and glycation regulation to also be of prime importance in defining individual fitness, and moreover in shaping species’ life-history. However, it is important to mention that the relationship between glycation and glycaemia is not systematic [36]. Indeed, high glycaemia levels may be beneficial for fitness (by relaxing energy-based trade-offs), while low glycation levels may be concomitantly selected as an anti-senescence adaptation. This may account for the lack of (e.g. interspecific) correlation between the two traits, while both are related to individual fitness. Hence, glycaemia and glycation seem to be proxies of individual fitness and not just labile variables that follow the nutritional status of organisms. This is probably owing to their central energetic role and, at the same time, to their chemical characteristics that, hypothetically, make them harmful molecules for fitness. What evidence do we have to suggest such an evolutionary role?

### (a) Glycation in relation to reproduction and survival

Fitness is optimized differently in different species, and local selection pressure has shaped specific trade-offs on life-history traits such as those related to reproduction and survival, thereby defining the pace-of-life of species [37]. Several studies have therefore tested the relationship between HbA1c levels and individuals’ reproduction in birds. In collared flycatchers (*Ficedula albicollis*), higher HbA1c levels correlated with increased adult reproductive success, namely bigger clutch size for females and higher number of fledged young for both males and females, after correction for laying date [38]. A positive correlation between HbA1c levels and growth rates of nestling has also been reported in American kestrels (*Falco sparverius*) [39]. Although the links reported are not proven to be causal, those two studies still suggest that high HbA1c levels reflect a higher investment in reproduction effort and growth. However, it should be noted that Récapet *et al*. did not find any correlations between HbA1c and other markers of reproductive success (i.e. probability to successfully fledge at least one offspring and number of total chicks) in collared flycatchers [40]. Still, they found a negative correlation between HbA1c levels and a proxy of survival (number of recaptures after a capture–marking–recapture protocol). According to the authors, these different results suggest that reproduction and growth, which both induce high metabolic demands, are life-history traits likely to trigger elevated glycation levels, ultimately leading to increased mortality rates. Thus, HbA1c, more than a simple variable that changes according to nutritional status, should be considered as a physiological variable that modulates individuals’ life-history trade-offs. However, those previous measurements of HbA1c levels in birds suffer from various methodological drawbacks (see Box 1), as discussed in Suo *et al*. [41] and in Brun *et al*., who showed no detectable HbA1c in zebra finches using a highly specific method [36]. It remains that little is known about the relationship between glycation levels and individual fitness, and that the few available studies solely focused on birds. Intra-population variability and heritability of glycation levels remain undefined in most animal species. Such data would enable us to assess whether glycation and the proteins (receptors, enzymes) involved in related regulatory pathways can be targeted through natural selection.

Box 1.
**Methodological issues related to protein glycation analysis in most studies involving non-conventional animal models**

To date, most studies measuring HbA1c in non-conventional animal models have been using chromatography methods [38] or commercial kits based on either boronate affinity [39] or cation exchange chromatography coupled to UV detection. Capillary electrophoresis has also been used by Oikonomidis *et al*. to quantify HbA1c in dogs. Concerning glycated plasma proteins measurements, competitive ELISA kits are the most used. AGEs, on the other hand, are often detected using fluorescence methods. However, all these methods lack specificity and sensitivity (see [41] for more detail). It should also be noted that commercial kits are targeted specifically at humans or laboratory mice and rats, which may induce an additional bias. Moreover, some studies performed HbA1c measurements on whole blood [39] instead of isolated red blood cells, with the result that other blood proteins most likely interfered with the final values measured. The gold standard method to measure glycation levels is mass spectrometry, as used by Brun *et al*. [36]. This methodology ensures specific glycation sites in proteins are targeted with high sensitivity.

### (b) Correlations between glycation and body mass

Among the different traits that determine the life-history of a species, body mass is one of the most important [42], because it allows interspecific allometric relationships among traits to be assessed as well as intraspecific variability in relation to individual quality. However, to our knowledge, only one correlational study in zebra finches addressed the links between glycation and body mass at an intraspecific level, and no correlation was found between plasma protein glycation levels and body mass [36]. On the other hand, some interspecific studies have been conducted on this subject. A recent work focusing on plasma AGE concentrations in seven birds and 16 captive mammal species of different body sizes showed that birds displayed higher AGE levels than mammals, but no significant correlation with body mass was detected in either class, even after phylogenetic correction [43]. Similarly, another study in Canidae failed to correlate plasma AGE levels with average body mass in each species [44]. An identical result was obtained when restricting the analysis to domestic dog strains, although dog breeds vary widely in body size [44]. This suggests that glycation is a variable likely reflecting a biological feature that stands above a simple body condition index, as it has been recently suggested for glycaemia [45,46], a hypothesis that needs a broader exploration at the inter- and intraspecific levels.

### (c) Glycation in relation to longevity

In humans, it is widely admitted that the levels of glycation-derived molecules (and more specifically of AGEs, the ultimate products) increase with age, thus taking part in the biological modifications that accompany the senescence process (e.g. loss of skin elasticity, crystalline opacification, etc.) [22]. Similarly, because glycation is associated with ageing-related diseases, it contributes to an increased probability of death. This is in accordance with the pro-senescent role of glycation and AGEs in humans, owing to the lack of selection pressure to set up any anti-glycation protection systems.

In non-conventional animal models, the picture is more mixed. The dynamic of glycation with age has been studied at an intraspecific level in a few species from different taxa. In mammals, while no correlation between age and HbA1c levels was found in the domestic dog (*Canis familiaris*) [47], a positive correlation between skin pentosidine levels (a specific cross-linking AGEs) and age was highlighted in seven mammal species (respectively least shrew, rat, domestic dog, cow, pig, squirrel monkey and rhesus monkey), with tighter relationships in shorter-lived species [48]. This is in line with the human observations. In insects, it was shown that older flies *Drosophila melanogaster* displayed 44% less AGEs than younger ones [49]. This may reflect a selective disappearance over time of individuals with higher AGEs levels, reinforcing the idea that the rate at which glycated products are accumulated may impact individual fitness.

When looking at the interspecific level, a negative correlation was underlined between the rate of formation of pentosidine in skin collagen and maximum lifespan in mammals [48]. This highlights that shorter-lived species accumulate pentosidine more quickly than longer-lived ones, which might be coherent with the fact that glycation accelerates actuarial senescence (e.g. the increase in mortality with age), and therefore might contribute partly to a shorter lifespan, both between and within species. Interestingly, in their extensive study of plasma AGEs levels in mammals and birds, Baker *et al.* found no correlation between age and AGEs levels in birds [43]. When focusing on 16 mammal species, after correction for phylogeny, they also did not highlight any correlation between age and plasma AGEs [43]. The absence of correlation between the plasma levels of AGEs or HbA1c and age was also observed at a smaller scale in wild canid species and domestic dogs of different breeds [44,47].

Taken together, these conflicting results suggest that there is not necessarily a direct link between AGEs accumulation and age. The dynamic of glycation with age is likely to be a species-specific trait, which might be largely dependent on (i) the metabolic adaptation to high sugar diet (i.e. which absolutely needs to be buffered for), (ii) the flying ability (see §4.1), or (iii) the methodological issues related to the type of tissue considered and on the nature of the glycated compound used as a marker of senescence. Indeed, we might expect to observe a more marked accumulation of glycation products in tissues with slow protein turnover rates [50]. Furthermore, there is a higher probability of observing an age-dependent accumulation of AGEs rather than Amadori compounds, as AGEs are stable over the long term owing to their (supposed so far) non-degradable status and difficult clearance. Conversely, Amadori compounds have short lifespans, as they are among the first products formed during the glycation reaction chain, and they are prone to undergo further reactions owing to their relative instability. They can also be degraded by specific mechanisms (see §4). It, therefore, seems more likely to observe Amadori compounds correlating with glycaemia in the short term (as observed in diabetic patients) rather than with age. However, a slight increase in their levels with age in the context of a longitudinal follow-up cannot be ruled out, given that glucose tolerance (i.e. the ability of an individual to efficiently regulate their blood glucose level and to avoid potentially harmful effects of high glycaemia through specific mechanisms) is known to decline with age [51].

Other important differences, such as the sex-dependent differences in glycation (see [47,52–54]) and their implication in sex–ageing patterns also need further examination. Longevity differences between males and females can be observed in many species, and one could expect the longest-lived sex to exhibit lower levels owing to their ability to prevent their early formation. We indeed already know that AGEs levels can differ between genders in patients with ageing-related diseases [52] or in pathological laboratory rats [54].

In conclusion, it appears that the present knowledge on how glycation might be associated with life-history traits and age in non-model species remains highly heterogeneous, and even contradictory in some specific cases, whatever the scale (inter- or intraspecific) or the parameter considered (see table 2). One possible explanation lies in the methodology of the studies carried out to date. They are all population-based and therefore cross-sectional, and it cannot be ruled out that the selective disappearance of the weakest individuals or those with the highest glycation levels does play a role in the heterogenous results obtained. Long-term studies with follow-up of individuals appear necessary to assess the possible links between glycation and age. It is also interesting to note that most studies looked at each life-history trait separately, although all these traits are intrinsically linked and influence each other. A global approach integrating the whole panel of traits defining the species’ pace-of-life and several markers of glycation at once is still lacking. Only such an approach might assess how glycation and life-history traits may have been shaped in relation to environmental challenges by evolution. Finally, we need more precise descriptions of the metabolic and cellular pathways through which glycation resistance may be acquired, and how such mechanisms may be related to higher individual fitness. Because (hyper)glycaemia levels are different among clades (i.e. mammals and birds), it is likely that the resistance mechanisms that emerged and their importance as modulators of fitness will differ among them.

**Table 2.** Known correlations between glycation products and age, glycaemia and/or life-history traits in non-conventional animal models at (*a*) intraspecific and (*b*) interspecific levels. + and - indicate positive and negative correlations, respectively, NS indicates no significant correlation.

glycation product	factor	correlation	species	comments	reference
(a) intraspecific studies					
HbA1c	age	NS	*Ficedula albicollis*	wild, adults	[38]
		NS	*Canis familiaris*	domestic, all ages, ANOVA on 3 age classes	[47]
	sex	NS	*Ficedula albicollis*	wild, adults	[38]
		NS	*Falco sparverius*	wild, nestlings	[39]
		NS	*Canis familiaris*	domestic, all ages	[47]
	body mass	NS	*Ficedula albicollis*	wild, adults	[38]
	clutch size	+	*Ficedula albicollis*	wild, adults	[38]
	number of offspring fledged	+	*Ficedula albicollis*	wild, adults	[38]
		NS	*Ficedula albicollis*	wild, adults	[40]
	arrival date on reproduction site	−	*Ficedula albicollis*	wild, adults	[38]
	return rate	−	*Ficedula albicollis*	wild, adults	[40]
	nestling growth rate	+	*Falco sparverius*	wild, nestlings	[39]
	number of brood mates	NS	*Falco sparverius*	wild, nestlings	[39]
GA	age	NS	*Taeniopygia guttata*	captive, adults	[36]
	glycaemia	NS	*Taeniopygia guttata*	captive, adults	[36]
TPGP	age	NS	*Taeniopygia guttata*	captive, adults	[36]
	sex	NS	*Taeniopygia guttata*	captive, adults	[36]
	body mass	NS	*Taeniopygia guttata*	captive, adults	[36]
	glycaemia	NS	*Taeniopygia guttata*	captive, adults	[36]
glycated serrotransferrin	age	NS	*Taeniopygia guttata*	captive, adults	[36]
	glycaemia	+	*Taeniopygia guttata*	captive, adults	[36]
glycated carbonic anhydrase	age	+	*Taeniopygia guttata*	captive, adults	[36]
	glycaemia	NS	*Taeniopygia guttata*	captive, adults	[36]
pentosidine (skin)	Age	+	*Cryptotis parva*	captive, 0–3 y.o.	[48]
		+	*Rattus norvegicus*	captive, 5–25 y.o.	[48]
		+	*Canis familiaris*	domestic, 2–14 y.o., beagle breed	[48]
		+	*Bos taurus*	captive, 0–14 y.o.	[48]
		+	*Sus scrofa*	captive, 0–15 y.o.	[48]
		+	*Saimiri sciureus*	captive, 3–21 y.o.	[48]
		+	*Macaca mulatta*	captive, 5–27 y.o.	[48]
(b) interspecific studies					
plasma AGEs	age	NS	mammals	captive (zoos)	[43]
		NS	birds	captive (zoos)	[43]
		NS	wild canids & domestic dog breeds	captive (zoos) and domestic	[44]
	age/MLSP	NS	mammals	captive (zoos)	[43]
		NS	birds	captive (zoos)	[43]
		NS	wild canids and domestic dog breeds	captive (zoos)	[44]
	body mass	NS	mammals	captive (zoos)	[43]
		NS	birds	captive (zoos)	[43]
		NS	wild canids and domestic dog breeds	captive (zoos) and domestic	[44]
pentosidine (skin)	MLSP	−	mammals	captive	[48]

GA, glycated albumin; MLSP, maximum lifespan; TPGP, total plasma glycated proteins (GA, glycated serotransferrin and glycated carbonic anhydrase).

## 4. Mechanisms of glycation resistance that need to be explored in a broad range of animals

The apparent resistance of bats and birds to high blood glucose levels may be explained by specific physiological and molecular mechanisms aimed at mitigating glycation stress. Interestingly, HbA1c levels measured in some bird and bat species showed lower levels than those of diabetic humans (e.g. 3.9% in the *Glossophaga soricina* bat [55], between 0.73 and 3.72% in collared flycatchers [40], no detection in zebra finches [36]), which supports the aforementioned hypothesis. Still, Baker *et al*. [43] found that AGEs levels are higher in birds than in mammals. This completes the resistance hypothesis by suggesting that resistance to high glycaemia can be reached at different levels (i.e. through early or late-glycation buffering). Thus, a more detailed exploration of the potential regulatory and/or resistance mechanisms in apparently high glycaemia tolerant animal species could lead to a better understanding of the glycation–life-history traits nexus.

### (a) Fast and effective glucose utilization: the case of flying vertebrates

Active glucose metabolism enables rapid glucose utilization and/or effective storage in cells immediately after food digestion, which can reduce the amount of blood glucose available for glycation reactions. Birds and bats both undertake active flight, which is one of the most energy-demanding activities that has evolved in multiple clades [56–59]. It has been shown in nectar- and fruit-feeding bats that flight is one of the key factors in post-prandial blood glucose regulation [55,60]. Studies on bats have shown that post-prandial blood glucose levels reach peaks that would be harmful in other mammals, but they drop quickly and reach fasting levels within 60–70 min after the bats spent 70–75% of their time flying [55,60], a behavioural-based mechanism that might almost replace insulin-mediated pathways in the *Glossophaga soricina* bat [55].

In addition to the above-mentioned behavioural adaptations (flight), physiological mechanisms are also primarily involved in glucose metabolism. Floral nectar is roughly composed of a 35 : 35 : 30% ratio of glucose, fructose and sucrose [61]. *Phyllostomoid* bats seem to rely on a well-developed arsenal of intestinal sucrases, which are responsible for the efficient hydrolysis of sucrose [62], and therefore, favour the rapid absorption of sugar molecules. Birds also seem to be adapted to high-sugar regimes, as the maximal enzymatic activity of sucrases was found to be up to two times higher in hummingbirds than in nectarivorous bats [62].

In bats, ingested sugars are usually directly consumed instead of being converted into fat and stored in adipose tissue or as glycogen in liver and muscles. This is notably the case for nectarivorous bats, both at rest and while flying and foraging [63–65]. To explain such a fast utilization of absorbed sugars, muscles would be the ideal compartment to study. To date, we only have data from some studies that highlighted that adult bats’ erythrocytes have a higher permeability to glucose than most mammals and birds, thanks to a rapid GLUT-1 mediated glucose transport [66]. This is even more remarkable because high permeability to glucose in erythrocytes is a feature usually only observed during fetal and neonatal life in mammals [67,68], except for humans, higher primates, small odontocete whales and bats, where it continues into adulthood [66]. Still, this metabolic particularity cannot be generalized to other sugars, as bats’ red blood cells permeability to fructose seems really low [66], which conversely might reduce exposure of erythrocyte proteins to fructose toxicity. The case of avian erythrocytes is very different from that in bats: studies on domestic geese showed very low permeability to glucose [69] and facilitated hexose transport seems to be almost absent in pigeons [70]. It should be noted that, unlike mammals, including bats, avian red blood cells are nucleated and possess functional mitochondria [71,72], thus making these more prone to be fuelled by fatty acids instead of glucose, a possible indirect mechanism of protection against glycation.

While the insulin-dependent cascade is conserved across most vertebrates [73], it differs substantially in birds [74]. Indeed, mammals heavily rely on GLUT-4 for insulin-induced glucose uptake into cells, but some studies mention the absence of the GLUT-4 transporter in birds [75,76], while others claim to have detected it [77,78]. One hypothesis to explain these conflicting results might be because avian genomes remain incompletely annotated owing to complexity. The absence of GLUT-4 could also be compensated by other transporters, such as GLUT-12, which appear to play a predominant role in few avian species [74,79,80]. Recent studies have also shown that the Slc2a4 gene encoding GLUT-4 had undergone changes under selective pressure in bat species relying on high-sugar diets in comparison to their insectivorous counterparts [81]. Moreover, it is estimated that bats heavily depend on intestine passive paracellular absorption, which constitutes over 70% of their total glucose absorption [82–84]. While this far exceeds what is seen in non-flying mammals [83,85–87], it is worth noticing that similar observations have been made in small birds [86–89]. Some authors suggested that this heightened reliance on passive paracellular transport might stem from the necessity to compensate for a proportionally smaller intestinal tissue mass compared to their overall body mass [86]. In the same vein but among non-flying vertebrates, marmosets, primarily gum-feeders with a smaller intestine relative to mammals of comparable size, also demonstrate a significant 30% paracellular glucose transport [90].

### (b) Protection of biomolecules

#### (i) Protein turnover

Protein turnover contributes to the maintenance of protein homeostasis, which can be defined as the preservation of the stability and functionality of the proteome. Several intracellular mechanisms mediated by the proteasome and the lysosome are involved in degrading structurally altered proteins to avoid their possible toxicity. Various studies in different animal models have demonstrated that the rate of accumulation of AGEs in proteins is positively correlated with their half-life, and therefore, negatively with their turnover rate [48,50,91,92]. One study suggested that enzymes and intracellular proteins with a rapid turnover may, therefore, be protected from high rates of glycation [93]. However, it is now widely admitted that senescence is accompanied by a loss of proteostasis, namely a decline in the synthesis of chaperones and a decline in the activity of the proteolytic systems [94], which could then partly explain the accumulation of AGEs in proteins and cells over life [22,95]. In addition, proteostasis itself does not escape glycation stress [96].

#### (ii) Specific amino acid composition and three-dimensional conformation of proteins

Protein glycation mainly targets lysine and arginine residues [97–100]. However, several studies demonstrated that not all lysines or arginines in the same protein are subject to the same glycation rates. For example, of the 44 amino acids in the alpha and beta chains of human HbA1c, only five appear to be potential glycation sites, namely three lysines and two valines [101]. This site specificity obviously depends on the amino acid composition of a given protein, but also and above all, on its multidimensional conformation, with theoretically glycatable amino acids being either exposed at the surface or, on the contrary, inaccessible because hidden. Thus, the lower number of lysines in chicken albumin and the fact that most of these residues are located in the inner part of the protein (only one lysine on the surface) seem to explain why it is glycated at a lower rate than bovine or human albumin (three lysines on the surface) when exposed to the same increasing concentrations of glucose [102,103]. Moreover, the presence of certain amino acids (such as aspartic acid or specific binding sites for phosphate or phosphorylated molecules) in the neighbourhood of the preferential glycation sites also seems to be of importance [104]. It is also interesting to notice that birds from the Psittacidae family, which includes parrots and parakeets, have more lysine residues than other birds, yet cases of diabetes have been reported in parrots [105].

### (c) Buffering glycated proteins and advanced glycation end-products

#### (i) Deglycating enzymes

Several defence mechanisms occur *in vivo* to regulate the accumulation of glycation products at different stages of the Maillard reaction. Although research in this area is still in its infancy, researchers have already identified several enzymes capable of detaching the sugar from the amine function of the fructosamine, thus reversing the formation of Amadori products and stopping the glycation reaction at its early steps [106]. To date, three major classes of deglycating enzymes have been described, which differ in their physiological role and their catalytic mechanisms: fructosamine oxidases (in fungi and bacteria) [107], fructosamine-3-kinase (in bacteria, mammals [107] and birds [108]) and fructoselysine-6-phosphate deglycase (in bacteria) [106].

#### (ii) Receptor for advanced glycation end-products

Originally known for binding AGEs [109], the receptor for advanced glycation end-products (RAGE) is now recognized as a multi-ligand receptor of the immunoglobulin superfamily [110]. It is expressed in various tissues [111] and is involved in inflammatory and immunological pathways [112]. RAGE is found under two main forms: transmembrane, the binding of which activates different signaling cascades, and as a free circulating form that can bind ligands without activating any pathway [113].

Among the AGEs capable of binding RAGE, preferential candidates include CML and hydroimidazolone [112]. The consequences of AGE–RAGE interactions at the membrane include a significant increase in reactive oxygen species (ROS) production, the activation of the NFκB transcription factor, an upregulation of inflammatory pathways, increased cell adhesion to the extracellular matrix, as well as facilitated AGEs accumulation [112,114–116]. Studies in diabetic patients have shown higher RAGE levels in the kidneys, particularly in the context of nephropathies, as well as in atheroma plaques of patients suffering from atherosclerosis [117,118]. A positive correlation between RAGE and HbA1c levels has also been highlighted [118]. In addition, genetically modified mice that do not express RAGE appear to resist ageing-related diseases associated with hyperglycaemia [119–122]. Furthermore, it is worth noting that soluble RAGE is suspected to mitigate the negative impact of AGEs by sequestrating them [123].

For a long time, RAGEs were believed to be unique to mammals, with birds lacking them, which appeared to be a potential explanation for birds' resistance to glycation [124]. An evolutionary study points out that RAGEs emerged with mammals while being lost in birds [125]. However, recent research indicates that some bird species may possess genomic sequences related to RAGE, thus leading to an emerging debate [126,127].

## 5. Concluding remarks and future prospects

Our review clearly outlines the critical lack of research on glycation in species other than humans or laboratory mice and rats. However, as we saw before, some taxa such as bats and birds seem to have evolved tolerance and/or resistance to high glycaemia and/or glycation, while having an extended lifespan relative to non-flying animals. Studying the mechanisms of tolerance to high levels of circulating glucose in bats and birds, notably in wild conditions (as captive conditions might not accurately reproduce and reflect what happens in nature), will likely put us on the trail to new treatments for diseases such as diabetes in humans. A better understanding of the nature of the mechanisms involved in glycation resistance will also help us to determine how evolution has led to develop tolerances to the adverse effects of high glycaemia and glycation, and how glycation patterns and life-history traits are intertwined between and within species. Indeed, we already know that glycation interferes with mechanisms involved in life-history trade-offs such as oxidative stress and telomere shortening [20,128]. Thus, studying glycation could provide key data for explaining senescence rate variability among vertebrates. In depth studies on the relationship between glycation and fitness are necessary, which will require long-term records of individuals’ life trajectories with repeated sampling over life. Furthermore, defining the rates of glycation accumulation over life should allow us to assess if glycation may be part of the process triggering organisms’ entry into senescence, as suggested by previous results [129]. Long-term studies of glycation dynamics at the individual level will enable us to gather key information on three major points in the evolution of glycation resistance. That is, (i) the inter-individual variability of glycaemia and glycation levels, (ii) the heritability of glycation levels (could there be a genetic transmission of this resistance), and (iii) the effect of glycation in determining individual adult phenotype, physiological performances and ultimately inter-individual variations in fitness through the evaluation of reproductive and actuarial senescence and lifelong reproductive success.

## Data Availability

This article has no additional data.
